# Multiatlas segmentation of thoracic and abdominal anatomy with level set‐based local search

**DOI:** 10.1120/jacmp.v15i4.4468

**Published:** 2014-07-08

**Authors:** Eduard Schreibmann, David M. Marcus, Tim Fox

**Affiliations:** ^1^ Department of Radiation Oncology and Winship Cancer Institute of Emory University Atlanta GA USA

**Keywords:** atlas segmentation, image registration, level sets, multi‐atlas

## Abstract

Segmentation of organs at risk (OARs) remains one of the most time‐consuming tasks in radiotherapy treatment planning. Atlas‐based segmentation methods using single templates have emerged as a practical approach to automate the process for brain or head and neck anatomy, but pose significant challenges in regions where large interpatient variations are present. We show that significant changes are needed to autosegment thoracic and abdominal datasets by combining multi‐atlas deformable registration with a level set‐based local search. Segmentation is hierarchical, with a first stage detecting bulk organ location, and a second step adapting the segmentation to fine details present in the patient scan. The first stage is based on warping multiple presegmented templates to the new patient anatomy using a multimodality deformable registration algorithm able to cope with changes in scanning conditions and artifacts. These segmentations are compacted in a probabilistic map of organ shape using the STAPLE algorithm. Final segmentation is obtained by adjusting the probability map for each organ type, using customized combinations of delineation filters exploiting prior knowledge of organ characteristics. Validation is performed by comparing automated and manual segmentation using the Dice coefficient, measured at an average of 0.971 for the aorta, 0.869 for the trachea, 0.958 for the lungs, 0.788 for the heart, 0.912 for the liver, 0.884 for the kidneys, 0.888 for the vertebrae, 0.863 for the spleen, and 0.740 for the spinal cord. Accurate atlas segmentation for abdominal and thoracic regions can be achieved with the usage of a multi‐atlas and perstructure refinement strategy. To improve clinical workflow and efficiency, the algorithm was embedded in a software service, applying the algorithm automatically on acquired scans without any user interaction.

PACS numbers: 87.57.nm, 87.57.N‐, 87.57.Q‐

## I. INTRODUCTION

Segmentation of organs at risk (OARs) is one of the most time‐consuming tasks in radiotherapy treatment planning, with manual or semi‐automated tools^(1)^ frequently employed to delineate OARs in a complex and lengthy process requiring frequent user interaction. Algorithms based on clustering,^(2)^ thresholding,^(3)^ or region growing^(4)^ are currently used for autosegmentation of clearly demarcated structures, but these algorithms commonly fail on organs with fuzzy boundaries, as voxels of similar intensity belonging to different organs are indiscernible to the algorithms' mathematics.^(5)^ Such vague boundaries are common in abdominal anatomy, where critical organs such as liver, spleen or kidney are grouped together and imaged with similar intensities, making their distinction cumbersome to current segmentation algorithms.^(6)^

Atlas segmentation^7^, ^8^, ^9^, ^10^, ^11^, ^12^ has emerged recently as an alternative to increase accuracy, compared to standard segmentation techniques, by incorporating both spatial information and voxel classification schemes into the algorithm's design. In a fundamental paradigm shift, the atlas‐based approach relies on the existence of a map between a reference image volume (called an atlas) in which structures of interest have been segmented and validated by an expert, and the image to be segmented, called the subject. A point‐to‐point map generated by deformable image registration transfers structures from the atlas onto the subject dataset. In clinical evaluation studies,^13^, ^14^, ^15^, ^16^, ^17^, ^18^ this approach has been shown to be superior to classical methods on structures that do not have a clear border, as their location is deduced from the atlas. The key element in this approach, however, is the accuracy of the deformable registration algorithm that is used to identify corresponding organs.

Due to its practicability,^(19)^ this method has recently been adapted in radiotherapy for segmentation of CT datasets of the brain^(16)^ or head and neck region,^14^, ^17^, ^20^, ^21^, ^22^ where interpatient anatomical variations are small and, thus, accurate mapping can be generated by the deformable registration. A wealth of techniques based on manual landmark‐based registration,^(23)^ on a BSpline^17^, ^24^ registration using a multimodality metric^(25)^ or a demons approach^(12)^ have been used to generate the correct map for these anatomic sites. For other sites, atlas segmentation has been proposed for clearly demarcated structures. Reed et al.^(10)^ use atlas segmentation for detecting breast boundaries in radiotherapy planning using the demons deformable registration algorithm, and Ehrhardt et al.^(26)^ apply the concept for identifying bones in preplanning for hip operations, using a global alignment with the aid of an affine transform, followed by a local matching of corresponding structures using the classical Thirion's formulation of the demons algorithm.^(27)^ Han et al.^(8)^ also employs multi‐atlases matched to the patient dataset in a multihierarchical registration using three stages of linear, nonlinear affine and shape‐constrained dense deformable registration with a specialized image metric that better aligns image edges, when compared to the classical mutual information. The technique has been reported for segmenting the bronchial plexus in lung cancer therapy by Yang et al.^(28)^ In general, systematic evaluations of the atlas segmentation concept for brain, and head and neck, anatomy in clinical practice have characterized the approach as a robust and reliable^(16)^ method for providing rapid delineation of target and normal tissues^(20)^ with a favorable balance of accuracy and robustness.^(29)^ Additionally, this method has been shown to decrease interobserver variability^17^, ^20^ with measurable consistency and time savings,^(13)^ reducing segmentation times by 35%^(17)^ to 63%.^(20)^

Although atlas segmentation techniques have been successful in anatomic regions with small interpatient variability, such as the brain or head and neck, we are not aware of any reports or commercial software applying the concept to segment OARs in regions with high interpatient variability. In our initial tests, using commercial and research software, significant discrepancies in accuracy have been observed between segmenting anatomy with low and high interpatient differences using this approach. These differences can be attributed not only to the high anatomical variations, but also to how the deformable registration would handle items that may be present in the subject patient, but not in the atlas such as implants, catheters, one lung versus two lungs, or pacemakers. In addition, variations in bowel content, various artifacts, and variations in image acquisition protocols frequently invalidate assumptions made in the monomodality deformable registration systems, including the classical demons approach.

The goal of this study was to design a new atlas segmentation system for thoracic and abdominal anatomy. Within the proposed approach, the system includes: a) use of a multi‐atlas approach to better identify organ shapes and locations,^(30)^ b) usage of a multimodality deformable algorithm capable of coping with variability in contrast and Hounsfield unit (HU) calibration,^(25)^ and c) a last step to refine the segmentation boundaries obtained in the multi‐atlas segmentation step to fine organ details,^31^, ^32^ while ignoring objects outside the initial segmentation. The spotlight of this work is creation of a first atlas system for abdominal anatomy with large intersubject variations by extending to extracranial anatomy an approach previously reported when segmenting brain or head and neck anatomy. In the following, we present our experience with algorithm design and parameter selection, and detail results on clinical datasets.

## II. MATERIALS AND METHODS

**Figure 1:**
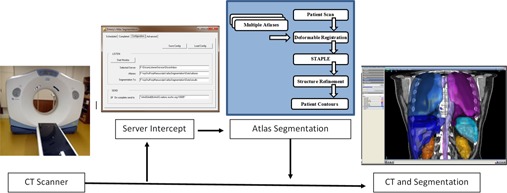
Workflow of the automated atlas‐segmentation approach. A dedicated server monitors files acquired on the CT scanner and starts the segmentation algorithm once a new dataset is detected. Once completed, results are inserted and attached to the CT scan in the clinical database through the DICOM protocol. The blue insert details steps of the proposed segmentation procedure. Multiple atlases are initially matched to the patient dataset using a deformable registration technique. The STAPLE algorithm is then employed to compact individual atlas result into a probability map of organ position and shape. This estimate is further refined using level set‐based segmentation.

A scheme of the overall procedure is presented in Fig. 1, detailing the input and algorithms used in our approach. First, a set of atlases are matched sequentially to the patient's CT dataset using deformable registration^(11)^ to produce a set of segmentations (see Materials & Methods section B). For each organ in the segmentations, the STAPLE algorithm^(33)^ is employed to deduce a most probable shape (Material & Methods section C). This estimate is then used as input to customized level set‐based algorithms^(34)^ (Materials & Methods section D) to produce a highly accurate segmentation capturing fine anatomical details.

### A. Datasets and server configuration

The end result of the proposed algorithm is a software module operating without any user interaction that automatically attaches segmentations of normal tissue to new CT scans. The images are monitored using a Digital Imaging and Communications in Medicine (DICOM) software listener, as shown in Fig. 1. When new scans are detected, the software automatically applies the proposed segmentation algorithm. At the end of the segmentation procedure, results are automatically inserted to the treatment planning system and the physician is notified of segmentation availability through an automated email. These results can be inspected and tweaked, if needed, using the standard tools available in the treatment planning software. In contrast to classical approaches, where intermittent user interaction is required, this algorithm completes the autosegmentation process independent of any interaction.

**Figure 2:**
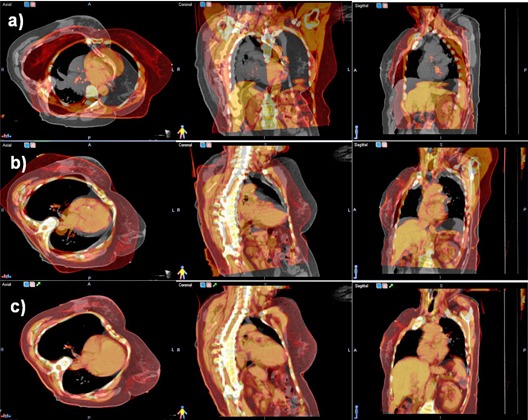
Steps of the atlas matching procedure show in axial, coronal, and sagittal planes, one atlas dataset (red color coding) superimposed on the patient dataset (gray color coding). In the initial match (upper row), significant differences between the atlas and dataset are observed as the patient lies differently in the scanner. The rigid match (second row) rotates and translates the atlas to match in orientation the patient dataset. The deformable registration (lower row) matches the external contour and the bulk of internal organs.

To achieve this degree of automation, the algorithm has to automatically deduce the anatomical site imaged, and to automatically correct for nonconformal patient positioning during the scan, such as a prone or lateral setups. To deduce the anatomical part imaged, we use specific DICOM tags to read the scanning protocol that is selected by the scanner operator according to the anatomical site. To correct for peculiar patient positioning, we use a two‐step initial alignment between atlases and patient dataset, where the first iterations solve translational differences, followed by a full affine registration that will rotate the atlas dataset, if needed, from the standard supine position to the scanning position used in the patient dataset. An example of such a clinical case scanned in a different posture is given in Fig. 2.

The user can override the automatically deduced setting to specify a set of atlases. For each anatomical site, three representative atlases of different body sizes are presegmented, but the user has the possibility to save additional atlases customized to his segmentation preferences or protocols. This is a practical trade‐off as, in principle, increasing the number of atlases leads to better accuracy at the cost of a longer computation times.

Datasets used for validation are all clinical scans acquired on the institution's scanner (LightSpeed RT 16; GE Medical Systems, Waukesha, WI) using the standard acquisition protocol for abdomen and thorax in a two‐month period while developing the proposed approach. All datasets were used for the validation, independent of patient size, acquisition protocol, patient positioning or treatment site. On our scanner, both abdominal and thoracic scanning protocols produce images of 2.5 mm voxel size, with a typical scan having 100 to 200 slices depending on the field of view size that was 40 cm for the abdominal and 50 cm for the thoracic scans.

### B. Atlas registration algorithm

Typical steps of an atlas segmentation procedure are illustrated in Fig. 2. The algorithm first matches each atlas to the patient dataset through a rigid matching, aimed to correct posture changes, followed by a deformable registration that matches internal anatomy.

The registration is a two‐step procedure, where we first use an affine transformation that corrects translations, rotations, and scales. The transformation is initialized by matching the center of mass for the gray scale values in the images in an optimization procedure that matches the images as judged by the Mattes formulation of the mutual information as metric.^(25)^ The transform parameters are found in an optimization procedure that uses a regular step gradient optimizer that iteratively changes the translations in incremental steps proportional with the changes in the cost function. Because only a few variables have to be optimized at this stage, we evaluate the metric on only half the number of voxels and use 20 histogram samples. In clinical practice, we observed that translations have a larger magnitude when compared to rotations that are restricted by the rigid patient support. Therefore, in the first 30 iterations, we favor translations to correct primordially for nonstandard patient positioning and other large variations in scanning positions, while in the further 30 iterations the optimizer settings are modified to consider shear, scaling, and rotations, in addition to translations, to further reduce interpatient variations.

The initial matching described above is followed by a deformable registration that switches the transformation from affine to BSpline,^(35)^ while keeping the same registration metric. The deformable registration uses the same concepts as the previous rigid registration, but increases accuracy by considering a local transformation that can model small changes in organ positions and shapes at the expense of a larger number of variables to be optimized. Indeed, the output of a deformable registration is a vector describing the translation of every voxel in the input dataset. Specific to the BSpline model, a practical trade‐off between speed and accuracy is presented by defining the deformation only in a few nodes overlaid on the input image, with the deformation in any location being interpolated from the nodes by basis spline functions. This approach has the advantage that only the deformation in the nodes is optimized, decreasing the number of variables to be found by the optimization procedure. As with the rigid registration, the optimization progresses under the guidance of a metric describing the match between the images to be optimized. In our setup, we choose to use the same Mattes formulation of the mutual information metric, but increase the numbers of samples, as a significantly larger number of variables have to be optimized.

To increase speed we use a common multiresolution approach, where the BSpline grid defining the deformation is progressively refined in four stages, starting from a course set of node spacing of 10 cm to a final spacing of 2 cm in the final stage. The optimizer in this stage is also replaced with the limited memory Broyden‐Fletcher‐Goldfarb‐Shanno (LBFGS) algorithm^(36)^ that approximates the gradient and has been found to be particularly suited to problems with very large numbers of variables.

In initial tests we noted that irrelevant regions have a large effect on the results decreasing accuracy in matching critical organs. Therefore, to eliminate irrelevant regions from metric calculation, the atlas segmentation with a margin is used as a mask for the moving image to compute the deformation only in voxels within defined organs. By this approach, the binary mask of the organs as defined in the atlas is expanded with a 1 cm margin, and used in the metric calculation to consider in calculations only voxels that mapped from the fixed to the atlas image would fall within the mask. All voxels that are mapped by the current transformation to nonrelevant regions are ignored.

### C. STAPLE algorithm

To adjust for variability among individual atlas results, the Simultaneous Truth and Performance Level Estimation (STAPLE) algorithm^(33)^ is employed as an algorithm that takes the collections of individual atlas segmentations results and calculates a probabilistic map of organ shape and location. The algorithm estimates the true segmentation by computing a conditional probability that minimizes discrepancies between atlas results.

**Figure 3:**
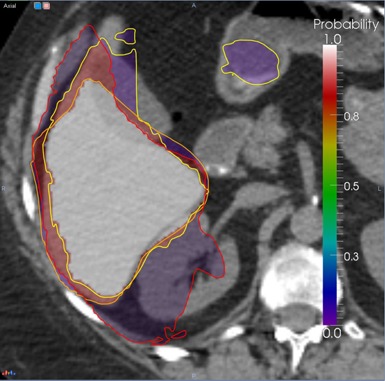
Deducing bulk organ shape from atlas segmentations with the STAPLE algorithm. The yellow, orange and red lines represent liver segmentations obtained by different atlases. The color overlay is the probability map that the corresponding voxel belongs to the liver as deduced from the individual segmentations, ranging from 0 (violet) to 1 (white).

The STAPLE algorithm is applied sequentially on all structures in the template, with the input being an organ's segmentation as obtained from warping atlases. The output of the STAPLE procedure assigns to each voxel in the patient's CT dataset a probability that that particular voxel is in within the segmented structure. As illustrated in Fig. 3, voxels that are encompassed in all segmentations are assigned a higher probability to be within the true organ boundaries, while voxels in just one segmentation are assigned a lower probability. In the example shown, the three templates used for the atlas segmentation of the liver did segment the general organ shape, but with many imperfections. Moreover, there was occasional leaking of the segmentation into nearby organs with similar contrast and HU values, as the algorithm attempted to reconcile the anatomical differences between the template and the atlas dataset. However, when combining the results obtained from each atlas together through the STAPLE algorithm, on generate a consensus from the atlases by identifying regions that are common to all atlases, that are assigned a higher probability of representing the liver and outlying singular results and marking them as improbable by assigning to them a lower probability of being part of the organ. The probability map ranges from zero to one and was thresholded in our experiments at a value of 0.7, determined empirically. As settings for the STAPLE algorithm we use 20 iterations. For generating the input to the refinement algorithm from the STAPLE algorithm, we threshold the map at a value of 0.7 that was deduced experimentally for the three atlases used as the region most probably covered by all atlases. Since the STAPLE is a maximum posteriory approximation, it can be, in principle, used as a representation of the level set in further developments of this algorithm if a larger number of atlases are used.

### D. Per‐structure refinement

**Figure 4:**
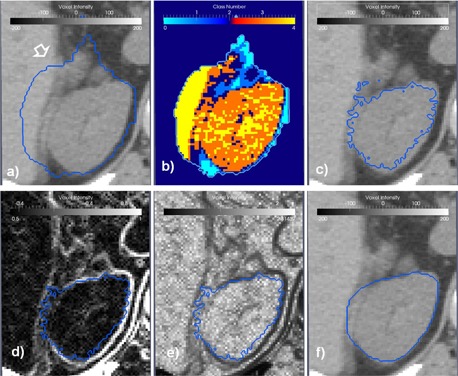
Steps of the refinement procedure: the input spleen segmentation is shown in (a) as a blue contour; (b) regions of different HU values are eliminated using a threshold level set‐based algorithm; (c) the shape is smoothed to image gradients using a geodesic level set algorithm.

As a final step, the organ shape approximated with the STAPLE algorithm is modified locally to smooth out the shape and adapt it to the fine details in the patient dataset using a level set‐based refinement technique. The level set technique was proposed in previous publications^37^, ^38^, ^39^, ^40^ as an efficient method to adapt an initial estimate of a shape to an image content. The classical formulation of the level set function has two competing terms — an image feature term that adapts the shape image features, while a second term keeps smoothness of the evolving shape. For example, a popular formulation of the image seeking term grows the shape according to the inverse of the image gradient to evolve faster on low gradient regions and stop at the edge of the organ. However, evolution under this term may leak on voxels displaying low contrast between structures, a fact that is contrabalanced by the smoothness term which maintains spatial integrity of the segmented region. Another popular image feature term includes in the evolution only voxels within a user‐specified threshold interval. These concepts and our particular implementation approach are detailed in Fig. 4.

The structure refinement technique is subdivided in our approach into three steps, where the result of every step serves as constructive knowledge for the next step. The main component of the refinement scheme is a threshold level set filter that adapts the shape to HU values within a user‐specified range, to distinguish the organ in question from other tissues. To make the refinement algorithm adaptable to different scanning protocols and the presence or absence of contrast media, this HU range is automatically extracted from the organ approximation obtained in the atlas segmentation procedure.

The initial shape as obtained from the STALE algorithm on the individual atlases is shown in Fig. 4(a) and includes the kidney, some parts of the liver, as well as some other soft tissue. Basically the proposed approach uses a threshold‐based level set refinement where the image term includes or excludes voxels from the initial term based on a specific HU units interval, followed by a gradient‐based level set optimization that aims to smooth out and introduce in the shape regions that might have altered HU values due, for example, to breathing motion. Additionally, organs to be identified show variable HU and noise profiles as determined by the scanning protocol and, thus, simple preset HU values for the threshold intervals would not work if a different scanning protocol is used. To this end, a classification procedure is used to detect from the images the optimal intervals for the HU thresholds. This is exemplified in Fig. 4(b), where an Otsu algorithm^(41)^ is used to classify the voxels within the initial shape in four classes. The colors in this figure shows assignment of each voxel to a class. The Otsu algorithm works by constructing a histogram of the voxels within the mask, and identifying in the histogram four peaks and the intervals that best divide the voxels classes by minimizing interclass variability. It is assumed that the class in most voxels represents the tissue to be segmented, while other classes represent adjacent tissue. A drawback of this approach is that the Otsu's threshold algorithm assumes a multimode distribution of the image that may fail to detect the sought object from background voxels if a poor contrast or uniform region presents, potentially leading to the level set diffusion refinement to create worse results. The following refinement step involves the use of the level set approach with an image‐based thresholding term to adapt the initial estimate to the expected shape based on the HU thresholds, as shown in Fig. 4(c). The last step in the refinement procedure aims to smooth out and include in the kidney segmentation the region marked with an arrow in Fig. 4(c) that has altered HU values due to respiratory motion. For this aim we use a gradient‐based image feature term to grow the shape. As with any level set function, first the image gradient is taken (Fig. 4(d)). The speed term that controls region growing is then taken as the inverse of the gradient image shown in Fig. 4(d) and is plotted in Fig. 4(e) with growing speed proportional with voxel intensity in this display. The result of this final step is shown in Fig. 4(f), where the resulting segmentation is smooth and conforms to the underlying kidney shape.

Technically speaking, options selected in our tests were a histogram of 125 bins that was divided in four classes by the algorithm. The minimum and maximum HU units for the subsequent level set‐based thresholding value were taken as the minimum and maximum HU value in the original image covered by the largest class, as classified by the Otsu algorithm. The shape was evolved for 500 iterations with a weighting term of 0.5 for the curvature term and a weight of 1 for the propagation term, and used the threshold formulation for the image term. The last step used the geodesic formulation^(42)^ of the image seeking term with a weight of 3 on the curvature to create a smooth‐resulting shape. The last step evolved for only 30 iterations to avoid leaking into nearby structures of same intensity or when the root mean square error change in the level set function is below $10e^{-5}$. These settings have been selected after extensive testing and, once established on the sample cases, have been used consistently for all cases and all organs included in the validation.

### E. Validation

We measured accuracy by comparing the degree of overlap between the automatic and manual contours using the classical Dice coefficient index,^(43)^ defined as the ratio of twice the overlap of two structures over the sum of their volumes. This coefficient is widely used in comparison studies being used in previous articles reporting accuracy from brain and head and neck segmentations, and has values ranging from 0, when the automated and manual segmentations do not overlap at all, to an ideal value of 1 denoting a perfect match between the two structures compared. To measure accuracy of the proposed method, validation through the Dice coefficient was performed retrospectively on 30 cases selected randomly from our patient database. The manually segmentations were all previously approved by an attending physician as part of each patient's radiation treatment planning process. For each patient, we applied the atlas segmentation procedure and compared the results with the manual segmentation using the Dice coefficient.

Complementary to the Dice coefficient, we also report the mean surface distance, which is another metric that measures the distance between the manually and automated segmentation for each point on the surface of the organ. This measure evaluates the distance between the manual and automated segmentation on a point‐by‐point basis, with the ideal value being zero when the two contours match. While the Dice coefficient measures the degree of overlap between the manual and automated segmentations, the Haunsdorf measure is valuable in detecting local discrepancies, such as spikes or local changes in the segmentation pattern.

## III. RESULTS

### A. Atlas registration algorithm

A typical result of deforming one atlas to the new dataset is illustrated in Fig. 2. The patient (first row, gray color scale) was scanned while lying laterally on the bed and, therefore, is tilted right as compared with the atlas (first row, red color scale superimposed on patient) that was scanned in the standard prone position. The rigid registration (second row) did correct for differences in posture and patient positioning by rotating laterally and moving down the atlas dataset, as compared to its original scanning location, but it was not able to match shapes and volumes of internal organs such as liver or lung. Results of applying the BSpline‐based deformable registration are shown in the last row, with the algorithm stretching the thoracic cage and liver volume in the atlas to match the appearance and shape of these organs in the patient scan.

### B. STAPLE algorithm

Usage of the STAPLE algorithm to factor out wrong segmentations is illustrated in Fig. 3, where liver segmentations obtained by three different atlases are shown as lines of different colors. None of the atlases was able to produce a clinically accurate segmentation, as one atlas undersegmented the organ, one included parts of the kidney, and one included parts of the bowel. The STAPLE algorithm can analyze these discrepancies to produce a probability map, shown as a colored overlay on the patient's CT dataset, with voxels marked violet having a low probability (5%) that the liver is present at that location as these voxels are included in only one segmentation, to voxels marked white being considered by the algorithm to be inside the liver, as these voxels are present in all segmentations. The miss‐segmentations of kidney and bowel were eliminated by the algorithm, as it assigned a very low probability to these voxels. The red regions have a probability of 95% of belonging to the liver, as these are voxels close to the high probability region, but not included in all segmentations.

Figure 3 also illustrate the necessity of using multiple atlases, as none of the single atlas results provided an acceptable clinical segmentation. Indeed, the Dice coefficient between the single atlas results and the manual segmentation for the dataset shown in the figure were 0.837, 0.838, and 0.831, respectively. The same coefficient between the STAPLE averaged atlases and the manual segmentation was 0.868. Similarly, the mean distances between manual segmentations and individual atlases were 1.177, 1.347, and 1.235 that decreased to 1.021 after merging the individual atlas results. The selection of an optimal number of atlases is an ongoing research topic with recent publications^44^, ^45^, ^46^ showing that a low number of atlases for STAPLE fusion may not produce robust fusion results. In the following we will use three atlases as input for the STAPLE algorithm in a trade‐off between speed and accuracy, opting for a lower accuracy in combining the individual atlases as the result is further processed by the per‐structure refinement.

### C. Per‐structure refinement

Usage of the refinement algorithm is demonstrated in Fig. 4 on a spleen, where the initial shape is shown as a blue line superimposed on an axial slice through the patient's CT dataset. Because of the proximity of the spleen to the stomach and their similar HU values, as well as a particularity in the patient's anatomy, parts of the stomach where marked as belonging to the spleen (as indicated by the arrow in the figure). This is a common misclassification in abdominal anatomy, especially in the regions where organs attach such as spleen‐kidney or kidney‐liver interfaces.

Such misclassifications are corrected in our refinement step by using sequences of segmentation filters customized for outlining soft tissue organs. The result of the level set‐based thresholding that preserves the appearance of the initial estimation is illustrated in Fig. 4(b), where the oversegmentation in stomach is eliminated by the algorithm. However, in this case, respiratory motion blurred voxel values at organ borders, modifying the expected HU units. To resolve this, a final step searches for the gradient of the structure from the threshold result, creating a final segmentation that is smooth and accurate.

### D. Validation

The atlas segmentation was configured to segment the following structures: aorta, esophagus, trachea, heart, lungs, kidneys, liver, spleen, and vertebrae. These contours were then compared retrospectively with clinical segmentations from a cohort of 46 patients randomly selected from our institution database. Not all clinical cases had all the atlas structures segmented, depending on the tumor location and clinical management. The statistics presented in this section includes five segmentations of the aorta, 13 of the esophagus, four of the trachea, 27 of the heart, 13 of the left and right lungs, 22 of the liver, 18 of the left kidney and 21 of the right kidney, seven of the spleen, and two of vertebrae.

**Figure 5:**
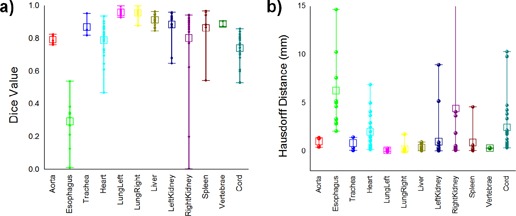
Box and Whisker plots for (a) Dice coefficients and mean surface distances, and (b) various thoracic and abdominal structures. The boxes show the 25th and 75th percentiles and the center line inside each box shows the median value, while the dots show the individual values.

**Figure 6:**
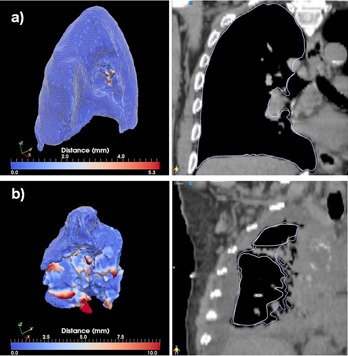
Best (a) and worst (b) cases of the right lung segmentations evaluated through the distance tool (left) and sagittal displays (right).

Comparison of Dice coefficients between the auto and manual segmentations is summarized in Fig. 5(a). For large and clearly visible structures such as the lung, very high conformity values are achieved, from 0.955 to 0.997 for the left lung and from 0.876 to 0.998 for the right lung. However, these differences are minimal, as illustrated in Fig. 6.

Large soft‐tissue structures such as the liver are matched at conformity between 0.846 and 0.963. As this is a large organ that significantly influences the registration's cost function, its location and shape is matched consistently by the deformable registration algorithm. As medium sized abdominal structures, the kidneys were matched at Dice values between 0.646 and 0.958 for the left kidney and 0.200 to 0.942 for the right kidney. For this organ, usage of a refinement procedure was important, as the deformable registration frequently distorts its shape in an attempt to reconcile anatomical differences between larger nearby organs. Even with the refinement algorithm turned on, the atlas segmentation may sometimes completely misplace this organ, creating incorrect segmentations, as documented by the low value of 0.200 obtained in one case for the right kidney. In practice, when validating the segmentations in daily clinical practice, such completely erroneous segmentations should be discarded and the organ should be resegmented manually. Similar to the kidneys, the spleen is matched at Dice values between 0.543 and 0.966, and the heart at values ranging from 0.832 to 0.935. The worst results are obtained for the esophagus, as it is a small structure of low contrast that has minor contributions to the cost function, with Dice values ranging between 0.010 and 0.538, accuracy not sufficient for clinical usage. A different segmentation approach may be needed to automatically segment this structure. The trachea was matched well at Dice value between 0.818 and 0.952, as this is an organ of high contrast compared to its surrounding anatomy. Accuracy measurements obtained with the alternative mean surface distance measure are summarized in Fig. 5(b), reflecting similar accuracy observations as noted in the Dice coefficient analysis. For structures that are matched well, such as the lung, the mean surface distance was less than 1 mm, between 0.097 and 0.339 for the left lung and 0.02 and 0.353 for the right lung. For the liver, the measure was between 0.087 and 0.983. Out of 19 segmentations of the left kidney, 17 displayed submillimeter mean surface distances (0.086 to 0.957), while two cases failed, recording values of 5.220 and 8.596 for this measure. Similarly, for the right kidney, out of 22 segmentations 17 had submillimeter mean surface distances, while the remaining inaccurate segmentations ranged between 1.915 and 41.555 mm. The worst match was obtained for the esophagus, in which all cases measured between 2.124 and 14.617 mm, confirming that utilization of this structure for autosegmentation in thoracic anatomy is impractical with the current algorithm. Vertebrae segmentation appears accurate by this measure, with values ranging between 0.292 and 0.413 mm; however, since only two comparisons could be made with manual contours, further evaluation of the accuracy of vertebral segmentation by this algorithm is needed.

**Figure 7:**
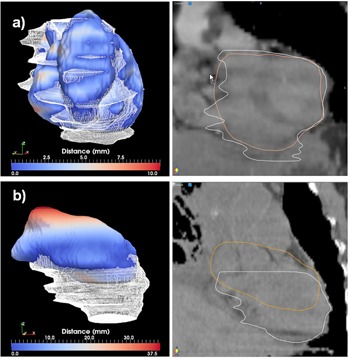
Best (a) and worst (b) cases of the heart segmentations evaluated through the distance tool (left) and sagittal displays (right).

**Figure 8:**
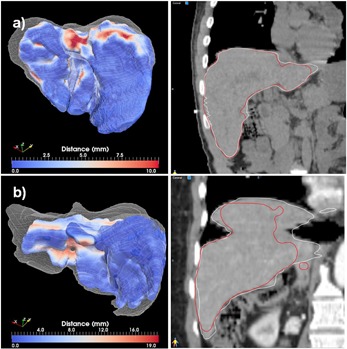
Best (a) and worst (b) cases of the liver segmentations evaluated through the distance tool (left) and sagittal displays (right).

**Figure 9:**
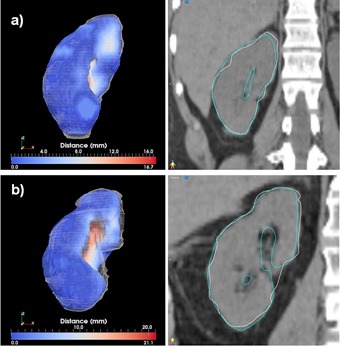
Best (a) and worst (b) cases of the right kidney segmentations evaluated through the distance tool (left) and sagittal displays (right).

**Figure 10:**
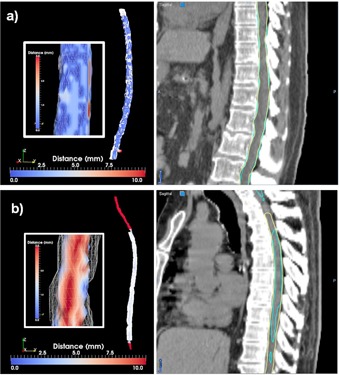
Best (a) and worst (b) cases of the cord segmentations evaluated through the distance tool (left) and sagittal displays (right). The insert in the left columns show a zoom‐in central region, with the scalar bar rescaled to show distances from 0 to 3 mm. The yellow contour in the right column shows the manual segmentation.

**Figure 11:**
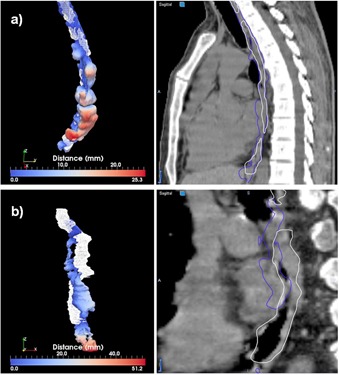
Best (a) and worst (b) cases of the esophagus segmentations evaluated through the distance tool (left) and sagittal displays (right).

The best and worst segmentations as measured through the Dice coefficient are shown in Figs. 6 to 11. In these displays, the wireframe represents the automated segmentation, while the color‐coded surface represents the manual segmentation. Color on the surface represents the distance in millimeters between the two segmentations. For the heart, the worst result is obtained for a case displaying a large lung tumor in the right lobe that shifted the heart estimation obtained by the deformable registration superiorly, leading to an incorrect positioning of the initial estimate. For the liver, the worst result is obtained on a case scanned with low resolution that resulted in an underestimation of the lung volume. For the kidney largest differences are observed on a case where the refinement did segment regions not included in the manual segmentation. For the lung, both automated best and worst segmentations display minimal differences from the manual contours. These cases demonstrate that variables, such as the presence or absence of tumor, the resolution of the scan, and the amount of overlap between scans, can significantly affect the quality of autosegmentation results.

Clinical accuracy cannot be achieved with the current setup for the esophagus, where the procedure cannot detect this organ with an accuracy that is useful in clinical practice. We also noted a worse performance on the right kidney over the left one, due to the presence of liver in close proximity to the liver that, as a larger volume, has a dominant influence over the deformation field at the expense of lower accuracy in the nearby organs. Additionally, kidney and liver are imaged with similar HU intensities, creating an additional hurdle for the registration metric and refinement procedure in discerning these organs. The relatively lower accuracy in the cord is explained by the different patterns of clinical segmentations and by the fact that, as an organ with smaller volume, segmentations errors have a higher influence on the Dice metric.

The clinical usefulness of this tool comes from reduction in the time needed for segmentation, as the autosegmented structures merely have to be modified, if needed, and validated, rather than segmented from scratch. Editing times vary by operator and the software used, but for a bulk estimate in our clinical operations these times are 4, 3 and 2 minutes, respectively, for structures having a Dice measure of 0.867, 0.942, and 0.960 for medium size structures such as the spleen. For larger organs, a typical liver segmentation matched at a Dice value of 0.92 was edited for inaccuracies in 2.5 minutes . On occasion, the automated segmentation failed significantly and, in such cases, it may be easier to just delete the autosegmentation and start from scratch, rather than trying to correct.

## IV. DISCUSSION

Most abdominal or thoracic organs cannot be autosegmented on CT images based on contrast and HU values alone, as many organs of similar intensity are located nearby or adjacent to each other. However, by warping a template of segmented structures to individual patient scans, these organs may be accurately incorporated into their associated regional context to create accurate autosegmented contours. In order to achieve this result, we constructed a segmentation technique that in a first step, can identify bulk organ position and shape and, in a second step, can recognize relevant details in the input datasets. The bulk estimates are an appropriate starting point for a segmentation technique based on level sets. In contrast to previous methods with voxel‐based approaches, we provide a technique that combines advantages of atlas registration and segmentation techniques to bring positional information into the segmentation process and subdivide the healthy media tissue into well‐defined OARs. The refinement step guarantees an important local closeness to distinguish organs from one another during the segmentation process.

In our initial tests, we observed that a single‐atlas approach gives suboptimal results, as larger volumes are correctly matched, but small structures lack clinical accuracy. For example, lung volumes are easily matched by the registration due to their high contrast from the surrounding tissue. In addition, the liver is preferably matched by the metric due to its size, despite low contrast with surrounding tissue. However for smaller organs, cross‐matching to other organs of similar HU values is frequent, with the kidney often partially matched to liver or spleen, or the aorta occasionally cross‐matched with heart or esophagus.

As shown in the Results section and illustrated in Figs. 5 and 7, despite these difficulties of the initial deformable registration, atlas segmentation can be customized and augmented into a viable segmentation approach for thoracic and abdominal anatomy if a postprocessing step adapting the atlas results to local organ features is constructed. With the level set‐based refinement procedure, the proposed automated method generates contours that are similar to the manual segmentations, but require significantly less user interaction.

Different approaches have been reported in literature to select the most appropriate atlas for segmentation, either using a representative average atlas^(47)^ or multiple atlases.^44^, ^48^ In the future, we hope to develop a selection algorithm that would select only atlases that show good anatomical matching as judged through the metric in the initial rigid alignment. Within this context, the refinement step at the end of the atlas‐based segmentation provides a method for decreasing calculation times, as we can use fewer atlases for the initial estimation of organ position and shape. As opposed to the previous approaches that have relied purely on the results obtained from the atlas‐based procedure, by blending the registration and segmentation procedures, the purpose of the deformable algorithm is not to obtain an accurate description of organ shape, but rather to detect its position as an initial estimate for the refinement procedure.

The method can also be extended to 4D CT datasets, where a higher level of noise is present, but possibly with a different approach to eliminate the noise by accounting for patient motion during acquisition,^(49)^ or with customized refinement settings to account for the increased level of noise in the 4D CT scans. Such extensions to the current algorithm to allow automated segmentation of multimodality images may be especially important in clinics implementing IGRT, where large datasets have to be segmented to track tumor changes or trajectory. For smaller clinics, the proposed method has the potential to significantly reduce the segmentation burden, and provides expert guidance for segmentation in the form of atlases that are presegmented by expert personnel.

## V. CONCLUSIONS

In this study, we tested modern segmentation techniques coupled with an atlas segmentation approach for high‐resolution segmentation of clinical CT images acquired for the purpose of radiotherapy planning. We found that for abdominal anatomy, usage of a multi‐atlas approach combined with refinement steps to capture small variations in organ anatomy is required. Validation results showed that our method produces accurate segmentation results even within the context of the variability in image shape and quality encountered in the course of standard image acquisition in the clinic. By integration with existing software, autosegmented contours are automatically attached to new scans and presented to the physician for review after the scanning procedure is completed. These methods have the potential to significantly improve the efficiency and accuracy of radiation treatment planning.
